# Primary Care Doctors’ Perspectives and Experiences With a Chest X-Ray AI Triage Program: Qualitative Study

**DOI:** 10.2196/103006

**Published:** 2026-09-17

**Authors:** Silin Kuang, Qi Wei Fong, Jacqueline Giovanna De Roza, Dana Hui Min Koh, Cher Heng Tan, Kai Ping Sze, Sabrina Kay Wye Wong

**Affiliations:** 1 NHG Polyclinics, Population Health Campus, NHG Health, 1 Mandalay Road308205, Singapore, 65 6355 3000; 2 Department of Diagnostic Radiology, Tan Tock Seng Hospital, NHG Health, Singapore; 3 Lee Kong Chian School of Medicine, Nanyang Technological University, Singapore; 4 Centre of AI in Medicine, Nanyang Technological University, Singapore

**Keywords:** artificial intelligence, chest X-ray triage, primary care, qualitative research, implementation research, technology adoption

## Abstract

**Background:**

AI has the potential to support chest X-ray (CXR) triage in primary care, but adoption depends on whether clinicians perceive its outputs as credible, useful, and workable within routine clinical workflows. Evidence on how primary care doctors experience AI-supported CXR triage in real-world practice remains limited.

**Objective:**

This study explored primary care doctors’ perspectives on a pilot CXR-AI program and identified barriers and enablers influencing adoption during early implementation.

**Methods:**

We conducted a qualitative descriptive study in a Singapore public primary care center where an AI system was embedded into the CXR workflow as a triage tool. Doctors who had used the program in clinical practice were purposively sampled across age, gender, and clinical seniority. Data were collected through semistructured in-depth interviews and focus group discussions, audio-recorded, transcribed verbatim, and analyzed using thematic analysis.

**Results:**

Twenty primary care doctors participated in 10 in-depth interviews and 2 focus group discussions. Adoption was variable and shaped by three interconnected themes: (1) AI validity and workflow integration, (2) clinician beliefs and confidence, and (3) organizational culture. Initial engagement appeared to be shaped by whether doctors understood the program’s purpose, perceived a need to change existing practice, and were open to workflow change. Continued use was shaped by the perceived accuracy of the AI tool and its usefulness in clinical practice. Doctors perceived the AI tool as more valuable when they were confident in CXR interpretation. Institutional endorsement, phased implementation, positive peer experiences, and the safety net provided by continued radiologist reporting helped build trust. However, concerns about AI overcalling, lack of clinical context and interaction, and medicolegal responsibility limited clinicians’ willingness to rely on AI alone.

**Conclusions:**

Adoption of AI-supported CXR triage in primary care depended not only on the technology itself, but also on how it was introduced, understood, and experienced in practice. These findings support the need for implementation strategies that are responsive to end user perspectives and contextualized within local workflows and clinical settings. Further research should examine later-stage implementation outcomes and objective operational and clinical outcomes of the program.

## Introduction

AI has shown promise in radiology [1]. In Singapore’s public primary care setting, chest radiographs (CXRs) are formally interpreted by radiologists, with a turnaround time of about an hour [2]. Against a backdrop of rising imaging demand and radiology workload [3], there is growing interest in AI solutions that improve efficiency in imaging workflows [1,4].

One such system is Lunit INSIGHT CXR (Lunit), a deep-learning AI system for CXR interpretation [5]. Lunit has been found to perform well in identifying normal CXRs, suggesting a potential role as a triage tool in primary care [6-8], where many CXRs do not show clinically important abnormalities [9]. In such settings, AI-supported CXR triage may improve operational efficiency, reduce radiologist workload, shorten reporting times, and support more timely clinical decision-making by primary care doctors [8,10,11].

In view of this potential, we implemented a pilot CXR-AI program in a Singapore polyclinic, a public primary care center with on-site radiography services. CXRs were triaged using Lunit, allowing primary care doctors to review AI-analyzed CXRs ahead of formal radiologist reporting [4,12]. To support its safe use in triage, we previously identified an operating threshold that prioritized sensitivity and minimization of false negatives. Lowering the threshold from the default 0.15 to 0.10 yielded a sensitivity of 93.2%, negative predictive value of 91.3%, and specificity of 71.7% [12].

However, as a newly implemented pilot program, it was unclear how primary care doctors would interpret, trust, and act on the AI outputs in daily practice. While existing studies have largely focused on diagnostic validation and perceived usefulness, fewer have examined feasibility and acceptability in real-world clinical workflows—key implementation outcomes that enable subsequent practice change [13]. Understanding how clinicians evaluate and respond to the new program is therefore important for optimizing integration into routine care and supporting sustained adoption [13-15].

In the broader literature, studies on clinicians’ perspectives of AI-enabled systems have identified factors such as perceived diagnostic accuracy, workflow and workload effects, medicolegal concerns, and regulatory oversight as shaping acceptance [16-18]. However, less is known about how these factors apply to AI-supported CXR triage in primary care, where their relevance may vary depending on clinical setting and workflow design [19,20]. Because adoption is a complex process rather than a single event [14], an in-depth exploration of clinicians’ real-world experiences is required to understand the factors shaping their adoption of the program in practice.

To address this gap, we conducted a qualitative study to explore primary care doctors’ perspectives and experiences with the CXR-AI program in daily practice and to identify the barriers and enablers influencing its adoption during the early implementation phase.

## Methods

### The Pilot CXR-AI Program

The CXR-AI program was rolled out in January 2025 in a single National Healthcare Group (NHG) polyclinic in Singapore. This polyclinic performs approximately 300‐450 CXRs per month. The AI tool, Lunit INSIGHT CXR, had received regulatory approval from Singapore’s Health Sciences Authority and underwent local validation and institutional review before implementation.

**Figure 1.**
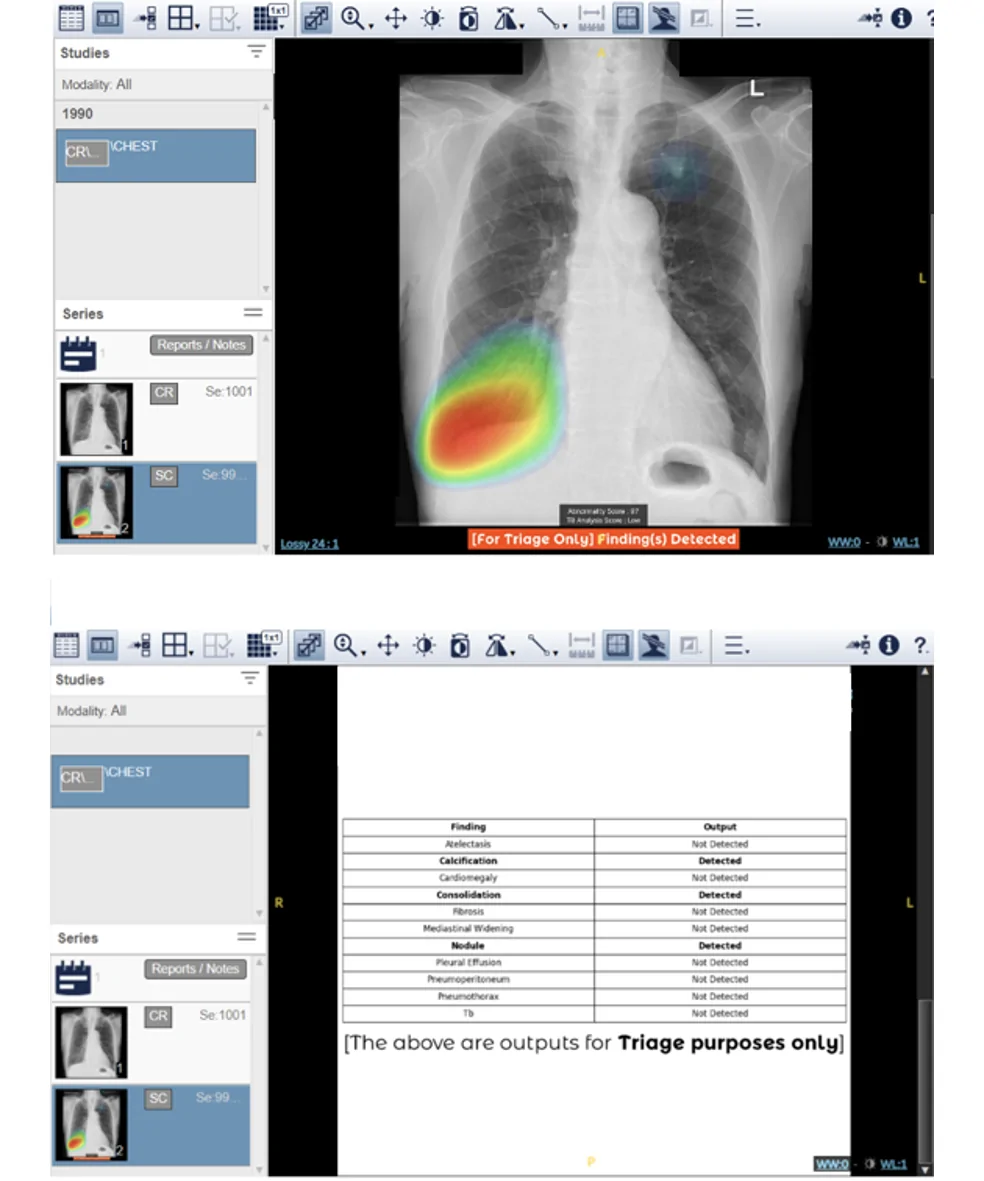
Lunit heat map and output table displaying detected abnormalities within the routine chest X-ray (CXR) viewing system.

**Figure 2.**
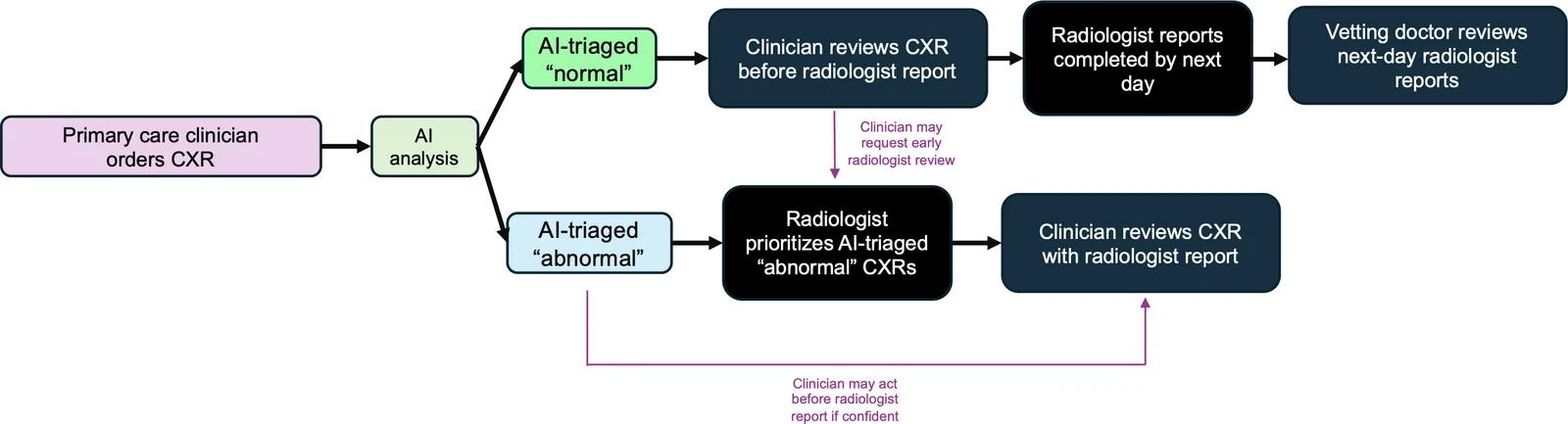
Overview of the CXR-AI program. CXR: chest X-ray.

In the CXR-AI program, all CXRs were analyzed by the AI tool, which displayed a heat map alongside a table of abnormality outputs within the routine CXR viewing platform (Figure 1). By default, CXRs triaged by AI as “normal” could be reviewed by the attending primary care doctor before radiologist reporting, allowing earlier clinical decision-making, and radiologist reports would be completed by the following day. In contrast, CXRs triaged as “abnormal” were prioritized for early radiologist reporting on the same day (Figure 2).

Clinicians could still override the default workflow. AI-triaged “normal” CXRs could be escalated for early radiologist review if the clinician was uncertain, while clinicians who were sufficiently confident could act on AI-triaged “abnormal” CXRs before radiologist reporting.

Regardless of AI triage status, all CXRs continued to undergo formal radiologist review. Radiologist reports completed the following day were reviewed by a vetting doctor, and patients initially discharged after AI-triaged “normal” CXRs could be recalled for further assessment if needed.

Between May 8 and July 18, 2025, a total of 587 CXRs were processed through the program, of which 69.8% (n=410) were triaged by AI as abnormal and 30.2% (n=177) as normal.

### Study Design

We used a qualitative descriptive design [21] to explore primary care doctors’ perspectives on using the CXR-AI program.

### Study Participants

Two months after program rollout, email invitations were sent to all doctors working at the polyclinic in which the CXR-AI program was implemented. Those who had used the CXR-AI program at least once in clinical practice were eligible for the study. We used purposive sampling to obtain maximum variation across age groups, gender, and clinical seniority levels, with selected doctors approached directly for participation.

### Interview Guide and Conceptual Frameworks

We developed the semistructured interview guide through iterative team discussions informed by relevant literature and three complementary conceptual frameworks (Textbox 1): Proctor’s implementation outcomes framework [13], the Theoretical Domains Framework (TDF) [15], and the Technology Acceptance Model 3 (TAM3) [22]. These frameworks were selected to capture early implementation outcomes, behavioral determinants of clinician use, and technology-specific adoption factors, respectively. Together, these frameworks guided the development of the interview prompts (Multimedia Appendix 1).

Textbox 1. **Conceptual frameworks and constructs informing development of the interview guide. The interview guide was pilot-tested with the first 3 participants to ensure clarity.**

**Proctor’s implementation outcomes**

Outcomes salient to early implementation:

- Acceptability
- Adoption
- Appropriateness
- Feasibility
- Fidelity

**Technology Acceptance Model 3 (TAM3)**

Independent constructs:

- Perceived usefulness (subjective norm, job relevance, output quality, result demonstrability)
- Perceived ease of use (computer playfulness, computer anxiety, perceived enjoyment, perception of external control)

Moderators:

- Experience
- Voluntariness

**Theoretical Domains Framework (TDF)**

Behavioral drivers:

- Beliefs about capabilities (perceived competence, self-efficacy)
- Beliefs about consequences (outcome expectancies)
- Professional role, identity, and boundaries
- Social influences (organizational commitment, group norms)
- Environmental context (environmental stressors, resources, organizational culture)
- Reinforcement

### Data Collection

We collected data using both in-depth interviews (IDIs) and focus group discussions (FGDs). Participants were assigned to either IDIs or FGDs based on participant preference, scheduling feasibility, and consideration of hierarchical relationships, with junior doctors not grouped with their supervisors. The same semistructured interview guide was used for both.

All IDIs and FGDs were conducted face-to-face in English, audio-recorded, and transcribed verbatim. IDIs lasted 45‐60 minutes and FGDs 60‐90 minutes. Sessions were held in private consultation rooms within the polyclinic, with no nonparticipants present.

The main interviewer (SK), a female family physician, conducted all sessions. A coinvestigator (KPS, QWF, DHMK, or JGDR) attended as an observer to document field notes, nonverbal cues, and group interactions. All researchers had prior training in qualitative interviewing. Although QWF oversaw implementation of the CXR-AI program and several investigators worked in the same polyclinic as the participants, none had supervisory relationships with them. All participants were aware of QWF’s involvement in the implementation of the CXR-AI program.

To support reflexivity, our team maintained reflexive memos and held regular discussions to consider how our clinical roles and prior assumptions might shape data collection and interpretation. The research team reflected on whether QWF’s presence might inhibit participants from expressing critical views of the program. To minimize this risk, QWF ceased attending interviews after two sessions. Participants were also reassured that their responses would remain confidential and were encouraged to share both positive and negative experiences. We conducted postinterview debriefs to reflect on emerging findings, consider whether researcher assumptions may have influenced question framing, and refine the interview guide for subsequent interviews.

Participants also completed a questionnaire capturing demographic and professional characteristics (Table 1).

**Table 1.** Participant characteristics.

Characteristic	Number of participants (%)
Age, years	
<30	3 (15)
30‐34	8 (40)
35‐39	4 (20)
≥40	5 (25)
Gender	
Male	13 (65)
Female	7 (35)
Clinical seniority	
Job designation	
Family medicine resident	6 (30)
Resident physician	3 (15)
Family physician	5 (25)
Associate consultant/consultant	6 (30)
Postgraduate qualification in Family Medicine	
Nil	7 (35)
Graduate Diploma in Family Medicine	3 (15)
Masters of Medicine in Family Medicine	8 (40)
Fellowship of College of Family Physicians Singapore	2 (10)
Work experience in primary care, years	
<5	9 (45)
5‐10	5 (25)
>10	6 (30)

### Data Analysis

Data were analyzed using thematic analysis following Braun and Clarke’s 6-step approach [23]. We employed both inductive and deductive coding, with codes primarily derived from participants’ accounts while being guided by the study’s conceptual frameworks. Data were managed in Microsoft Excel (version 16.78.3).

Each transcript was independently coded by two investigators (SK, KPS, QWF, DHMK, or JDR), and discrepancies were resolved through discussion. Codes, subthemes, and overarching themes were iteratively developed and refined through regular team meetings and organized into a coding tree. Emerging interpretations were continually checked against the transcripts and participant quotations during team discussions. We maintained an audit trail documenting coding decisions, theme development, and codebook revisions [24]. To examine whether the data collection method influenced the findings, we compared the representation of subthemes across IDI and FGD transcripts. We also examined whether the representation and emphasis of subthemes varied across participants with different demographic and professional characteristics.

Data collection and analysis proceeded concurrently. We ceased recruitment after 20 purposively sampled participants once the research team determined that the dataset provided sufficient depth and variation to address the research question. The remaining eligible doctors were not recruited.

To enhance trustworthiness, we used data triangulation, reflexive journaling, and member checking by returning transcripts to participants for verification and by clarifying interpretations with them during analysis [24]. Reporting followed the Consolidated Criteria for Reporting Qualitative Research (COREQ) guidelines (Checklist 1).

### Ethical Considerations

Ethics approval was obtained from the NHG Domain Specific Review Board (reference: 2024‐3771). All participants provided informed consent. Pseudonyms were used during FGDs. Recordings were transcribed and deidentified before analysis, and participant demographic data were deidentified before compilation. Each participant received a token of appreciation (SGD $30 [US $23.68]).

## Results

There were 31 primary care doctors eligible for participation at the study site. Over a 9-month period from March 2025 to November 2025, a total of 20 were purposively recruited, all of whom agreed to participate. We conducted 10 IDIs (A1-A6, D1-D4) and 2 FGDs with 5 participants each (B1-B5, C1-C5). Participant characteristics are described in Table 1.

**Figure 3.**
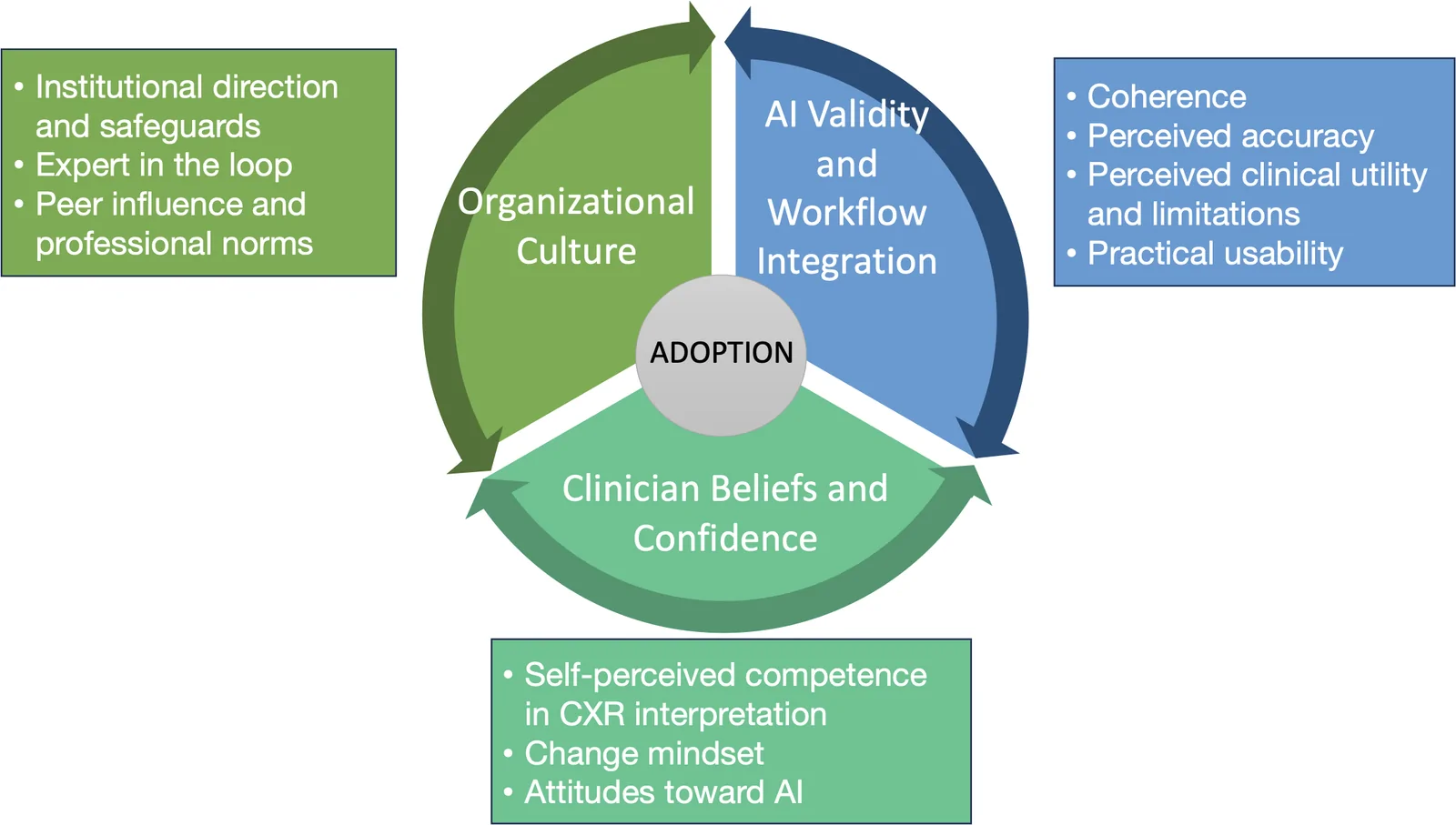
Primary care doctors’ perspectives of the CXR-AI program. Three interdependent themes shaped the adoption of the program. CXR: chest X-ray.

Three interconnected themes emerged from the analysis: (1) AI Validity and Workflow Integration, (2) Clinician Beliefs and Confidence, and (3) Organizational Culture. These themes collectively shaped clinicians’ perceived value of the CXR-AI program and subsequent adoption patterns (Figure 3). No subthemes were unique to either IDIs or FGDs. No clear patterns in subtheme emphasis were observed according to participants’ demographic or professional characteristics. Some factors appeared more salient when clinicians described their initial willingness to try the tool, while others became more prominent in their accounts of continued use.

### Theme 1: AI Validity and Workflow Integration

Adoption was shaped by clinicians’ understanding of the tool’s purpose (coherence), perceived accuracy, clinical utility and limitations, and its impact on workload.

#### Coherence

At initial exposure to the CXR-AI program, clinicians’ understanding of the AI tool’s purpose shaped their willingness to engage with it.

Those who perceived a clinical or operational problem, such as delays in radiologist reporting, were more likely to view the program as relevant and worth trying. As one clinician described, “on busy days...the radiographs can take two, three hours to report...there’s always this fear that what if it’s something that is urgent and needs to be seen faster?...that to me was a problem, and [the AI] has helped to fill in the gaps” (IDI: D4, 30, male).

In contrast, some clinicians saw no need to alter their current practice: “I don’t see any need for a workflow change from what I’ve been practicing” (FGD: C1, 33, male). Some viewed current reporting times as acceptable—“an hour and a half wait...not too bad for polyclinic” (FGD: C2, 42, male)—and therefore questioned the rationale for the program. Others expressed the need for clearer justification from the institution: “I need the institution to tell us that they are going to do something. What’s the purpose of doing it?...instead of just telling us that it’s probably going to be mainstream care” (FGD: C3, 36, male).

These divergent views reflected differences in clinicians’ perceptions of the appropriateness of the intervention. Based on participants’ accounts, coherence appeared important in shaping clinicians’ initial willingness to engage with the tool. With experience, participants’ continued engagement appeared to be shaped more by their evaluation of the tool’s accuracy and clinical utility in practice.

#### Perceived Accuracy

Once coherence was established, the perceived accuracy of the AI tool shaped trust and clinicians’ willingness to act on its outputs. Confidence arose when AI outputs aligned with their own readings or radiologist reports. One doctor found it “largely credible...[as it] correlates with what we expect” (IDI: A4, 36, male).

Clinicians also wanted evidence of the AI tool’s validation. As one explained, “For the AI tool to gain my trust, it has to be assessed...the same standards applied to it as any investigation...what’s the sensitivity, what’s the specificity, what’s the gold standard we are comparing against?” (FGD: C5, 32, female). Briefings on the AI’s performance data helped to reassure some: “There was a briefing...sensitivity rate was quite high. Having that knowledge in mind provides me mental reassurance that it’s most likely to be normal when it’s reported normal” (IDI: A6, 27, female). These accounts suggest that clinicians’ assessment of the AI tool’s accuracy influenced their confidence in acting on its outputs.

#### Perceived Clinical Utility and Limitations

Clinicians’ willingness to use the AI tool hinged on whether it was “useful” to their work and patients (IDI: A5, 46, male). Clinical utility centered on time savings, workflow efficiency, and complementing clinical decision-making.

##### Usefulness of AI-Triaged “Normal” CXRs

For many, the AI’s usefulness was most evident in triaging normal films: “What I like was that if there’s clearly a normal chest x-ray, we can discharge the patient much faster” (FGD: B1, 33, male). However, some cautioned that if there were AI errors not picked up by clinicians, “the patient may need to come back...but that will be a minority of patients” (IDI: A3, 30, female).

##### Usefulness of AI-Triaged “Abnormal” CXRs

In contrast, for AI-triaged “abnormal” CXRs, perceived usefulness was more variable and depended on whether clinicians were willing to act on the AI output before radiologist review in order to expedite care. Some appreciated it as a “second pair of eyes” (FGD: B1, 33, male) to confirm their own interpretations, particularly for clear-cut findings like consolidation in suspected pneumonia. However, because the AI operating threshold prioritized sensitivity, clinicians felt that it “tend[ed] to overcall certain things” and therefore preferred to wait for radiologist reports (IDI: A1, 38, male).

Clinicians also found the heat map useful for localizing single abnormalities: “the heat map generally is quite good because it tends to focus your attention on where it thinks the abnormalities are” (IDI: A1, 38, male). However, when multiple areas were highlighted, interpretation became more difficult: “*...*so many pathologies, the whole heat map lights up then I’m scratching my head...I cannot make [a] decision*...*” (IDI: A4, 36, male).

##### Perceived Limitations of the AI Tool

Clinicians highlighted limitations of the AI tool, including its inability to incorporate clinical context and to compare with prior CXRs. As one explained, “If [the consolidation has] improved, the AI is not going to help me because it’s just going to highlight the area as abnormal...there’s no interval comparison” (IDI: A3, 30, female). They also described the lack of interaction with the AI compared with a radiologist, with whom they can discuss clinical context and suspicions (IDI: A2, 41, male). One added that “if I can have that [discussion] with the reporting from the AI, that may perhaps give me a bit more confidence” (FGD: C1, 33, male).

##### Indirect Benefits Through Faster Radiologist Reporting

Regardless of how clinicians responded to the AI outputs, many observed indirect benefits such as faster radiologist reporting. They noted that “radiographs are being reported faster these days, and I like to think that may be because of the triaging process” (FGD: B4, 30, male), which in turn enabled doctors to “manage [patients] faster, whether they need to be seen in the emergency or...can be sent home” (IDI: D4, 30, male).

##### Contextual Limitations in the Polyclinic Setting

Some clinicians felt the AI’s clinical utility was modest in the polyclinic context, where CXRs form only a small part of a high-volume general practice workload. One explained that when doctors were busy attending to other patients, “the X-ray is not the limiting factor...in terms of [patients’] waiting time” (IDI: D1, 29, female). Another reflected that “it’s not that much of an impact because maybe I do two CXRs in the 40-something patients that I see [in a day]” (FGD: B5, 30, male).

### Practical Usability

Ease of use was frequently cited as “one of the biggest factors [influencing] adoption” (IDI: A5, 46, male). The tool was praised for its user-friendly interface and easy access within the electronic medical system. “It loads fairly quickly...the features are quite clear. The fonts are big. The heat map is staring at you. Interface is fairly straightforward...just a quick scroll and you can see the next image” (IDI: A4, 36, male).

### Theme 2: Clinician Beliefs and Confidence

Clinician beliefs and confidence refer to individual-level determinants of engagement with the AI tool. Three key factors emerged: self-perceived competence in CXR interpretation, attitudes toward AI, and change mindset.

#### Self-Perceived Competence in CXR Interpretation

Perceived value of the AI tool appeared related to clinicians’ confidence in interpreting CXRs and acting before radiologist reporting (IDI: A3, 30, female). For those already accustomed to doing so, the AI was a useful adjunct that accelerated patient care (IDI: D2, 57, male). For these clinicians, “the biggest advantage [of the AI] is that it’s much faster for the patients...I would definitely use it” (IDI: A5, 46, male).

In contrast, when clinicians felt less confident in interpreting CXRs, they preferred to wait for the radiologist’s report. “I think if we were to base the management of the patient purely from my personal reading of the image [and the AI output], I’m worried that may potentially be wrong, and then it may also have medicolegal consequences” (FGD: C4, 32, female).

#### Change Mindset

Clinicians’ openness to change influenced their willingness to try the AI tool even before its value in practice was fully established. One shared that since “it’s a new initiative, just try [it]. I keep an open mind” (IDI: D1, 29, female). Those who viewed change as an opportunity for improvement were more receptive to experimenting with new tools, describing this as dependent on the “personality and the working style of a clinician” (FGD: B4, 30, male).

In contrast, some clinicians were reluctant to stray from established workflows when they perceived no clear benefit. One admitted, “I probably would not have chosen the AI...Simply because I’m resistant to change, just my personality...I don’t see any need for a workflow change from what I’ve been practicing” (FGD: C1, 33, male).

#### Attitudes Toward AI

##### Beliefs About AI’s Future Role in Health Care

Clinicians expressed optimism about the AI’s potential and therefore accepted its use in its current form, expecting it to improve as the system matures. As one explained, “I think there always needs to be a take-off point somewhere for these innovations to happen” (FGD: B5, 30, male). Another added, “there will be a point in time where the AI output and the radiologist output will be...no significant difference. And the AI might be able to interpret it better” (FGD: C3, 36, male).

Others accepted AI integration as inevitable, reflecting that “AI has permeated through our lives.... If you reject it, then you are kind of left behind.... Since I can’t change the tide...I have to learn how to embrace and accept it*...*” (IDI: A4, 36, male).

##### Prior Exposure to AI

Prior experience with other AI systems may influence clinicians’ comfort with the AI tool. Those familiar with AI felt more comfortable, knowing “what are the thresholds, what are the limitations” (IDI: A1, 38, male), while others felt such experience was not transferable, as they “don’t use those for CXR” (IDI: A3, 30, female).

### Theme 3: Organizational Culture

Organizational culture captures how institutional structures and professional norms shaped clinicians’ engagement with the AI tool. The most prominent influences were institutional direction and safeguards, followed by the reassurance of an expert in the loop, and the reinforcing effects of peer influence and professional norms.

#### Institutional Direction and Safeguards

##### Institutional Endorsement and Regulatory Oversight

Clinicians’ trust in the CXR-AI program stemmed from their confidence in institutional governance and its framing as an institution-led initiative. For some, institutional support was a prerequisite for acceptance: “for this to be rolled out, I would need to know that there’s institutional support” (FGD: B2, 41, female). Institutional endorsement therefore conferred legitimacy on the AI tool. One explained, “it seems more credible when the whole institution has put weight behind it,” as he trusted that “they would have put in a bit more safeguards” (IDI: A5, 46, male). Another added that “especially in the government institution, we will put a lot of priority in terms of safety” (IDI: A4, 36, male).

Many also assumed that rigorous validation and regulatory review had been undertaken. Clinicians articulated expectations of what regulatory oversight should entail, including the AI system being “tried and tested independently, by an independent body outside the organization...[with] a process that continually audits this whole system” (IDI: A2, 41, male) and reviewed across multiple levels, including ministry and professional bodies (IDI: A4, 36, male).

##### Stepwise Implementation and Feedback Channels

Participants appreciated the phased introduction of the CXR-AI program, which built familiarity through briefings, reminders, and a “testing period” where clinicians could observe AI outputs while radiologist reporting continued as usual. “A stepwise introduction is important...to have that time to prove to the people who are using it...that this is a reliable tool” (FGD: B3, 28, female).

Although clinicians generally trusted institutional leadership, some felt implementation was largely top-down. One noted that “in our culture...if higher ups say ‘do this,’ we do this...nobody [asks] ‘why?’” and suggested creating opportunities to provide input before implementation (FGD: C3, 36, male).

Together, these accounts suggest that institutional authority both facilitated clinicians’ acceptance of the program and shaped their expectations of how it should be implemented.

### Expert in the Loop

Clinicians’ acceptance of the CXR-AI program was contingent on the continued involvement of radiologists. They felt “protected...that [all] x-rays will ultimately be reviewed by a radiologist...it’s a safety net for all of us” (IDI: A6, 27, female). Knowing that “the radiologist will still double-check,” clinicians felt comfortable to *“*trust [the AI] as kind of like a first triage” (IDI: A4, 36, male).

Beyond patient safety, clinicians valued the shared medicolegal accountability afforded by radiologist involvement. The prospect of removing radiologist reporting was deemed unacceptable because “100 percent of the responsibility will then fall upon the family doctor.... I wouldn’t like that at all. I think that’s quite unfair” (IDI: A3, 30, female).

Across interviews, radiologists remained the gold standard against which the AI’s value was judged, and their endorsement further strengthened its credibility. As one participant explained, “Whatever we learn and interpret, [the radiologists] had taught us in the first place. So, if they realize that the AI is quite trustworthy and credible, it boosts the credibility further” (IDI: D1, 29, female). In effect, the CXR-AI program was accepted not because of the AI alone, but because it was embedded within a workflow in which radiologists remained involved.

### Peer Influence and Professional Norms

Clinicians described how their colleagues’ behavior could shape their willingness to use the AI tool. One explained that “as human beings, there’s this herd effect...if many people are not using it, I might probably be influenced not to use it” (FGD: B2, 41, female). Another felt that “if the rest of the team feels that it is useful, then it lends more credibility and I want to use it more” (IDI: A5, 46, male).

Peer experiences were generally favorable, with clinicians sharing positive examples of the AI’s performance and how these had built their own confidence in the tool. One recalled hearing that “it picked up pneumothorax...then it escalated...then the patient received care” (IDI: D1, 29, female).

Beyond peer influence, clinicians linked professional norms to medicolegal reassurance, noting that the AI tool alone was not yet defensible in the event of errors. As one explained, “if it is something that’s recognized as of high standard and it is widely used, then it can help to protect you...in terms of medicolegal implications. However, if it is a process that’s under trial, definitely it will not be helpful in any defense” (IDI: A2, 41, male).

However, some clinicians cautioned that peer influence and practice culture should not determine adoption decisions, as professional responsibility ultimately rests with the individual doctor: “Just because it’s something that is widely used or accepted doesn’t necessarily mean that it’s correct.... Ultimately, I’m the one that has to call the patient back and spend time and effort to change the plan” (IDI: A3, 30, female).

Together, these accounts suggest that peer influence helped build confidence in the AI tool, but did not override clinicians’ sense of individual responsibility for decision-making.

## Discussion

This qualitative study provides insights into primary care doctors’ perspectives during the early implementation of a pilot CXR-AI program. Clinicians were broadly receptive to the technology, but their engagement was conditional, context-dependent, and shaped by hands-on experience.

### Determinants of Initial and Continued Adoption During Early Implementation

Different factors appeared more salient when participants described initial uptake versus subsequent use.

At the point of deciding whether to try the AI tool, coherence and change mindset appeared particularly important in participants’ accounts. Coherence refers to the sense-making work clinicians undertake to understand an intervention’s purpose and relevance to their practice [25] and overlaps with Proctor’s construct of “appropriateness” [13] and TAM3’s “job relevance” [22]. Some clinicians described being more willing to engage when they understood the purpose of the intervention and saw it as meaningful to their work. Similarly, a change mindset appeared to contribute to early willingness to try the tool despite uncertainty [26]. This is consistent with prior work showing that individual mindsets shape receptivity to health care innovations [27].

Following initial use, decisions about whether to continue using the tool were more influenced by clinicians’ assessment of its usefulness in clinical practice. This aligns with TDF’s construct of reinforcement, whereby behaviors are more likely to be continued when they produce rewards that are perceived as valuable and immediate [15]. In our study, these rewards took the form of diagnostic accuracy and workflow efficiency, which have also been identified in the literature as important preconditions for AI adoption [16,17]. The operating threshold used by our AI model provided high sensitivity (93.2%) and negative predictive value (91.3%), supporting its role in triaging normal CXRs, but lower specificity (71.7%) and positive predictive value (76.7%), consistent with clinicians’ perceptions of overcalling abnormalities [12]. These findings reinforce the potential role of Lunit in triage while highlighting its limitations as a stand-alone diagnostic tool [6-8,28,29].

### Context-Specific Findings: Institutional Legitimacy and Muted Medicolegal Concern

Two findings differed from much of the existing literature. First, institutional endorsement emerged as an important enabler of adoption. TAM3 conceptualizes voluntariness as a moderator of social norms’ effects on intention to use, such that in mandatory contexts, individuals may adopt a system due to compliance rather than internalized beliefs about its usefulness [22]. However, in our study, many participants appeared to interpret mandatory implementation not only as something to comply with, but also as a signal of legitimacy. Relatedly, whereas prior studies have described regulatory uncertainty as a source of anxiety [30,31], our participants generally expressed confidence in institutional oversight. This trust in institutional governance may reflect contextual factors specific to the local health care setting. While this could be influenced by broader cultural tendencies, such as a higher power-distance index [32], our study was not designed to examine these explanations directly, and such interpretations should be made cautiously.

Second, although medicolegal concerns are frequently cited as a major barrier to clinicians’ acceptance of AI [30,31,33,34], these concerns appear mitigated in our study. This may be because radiologists remained embedded in the workflow, preserving shared responsibility and reducing anxiety about liability. In the local primary care context, formal radiologist reporting of CXRs is routine [35], and clinicians are accustomed to practicing within this arrangement. Participants regarded radiologists as a crucial second layer of review because of their specialist expertise and greater exposure to CXRs. This is supported by prior studies showing that CXR interpretation by nonradiologists is associated with higher rates of diagnostic error [36]. Together, these factors help explain why the AI tool was viewed as a complement to, rather than a replacement for, radiologist reporting.

### Strengths and Limitations

A key strength of this study is that it examined clinicians’ perspectives after real-world use of an AI-supported CXR triage program embedded in routine primary care practice. This contrasts with much of the existing literature, which has explored AI acceptance in hypothetical settings. This is important as first-hand experience significantly shapes clinicians’ perceptions [26,30,37]. The use of multiple conceptual frameworks, together with both IDIs and FGDs, also strengthened the analytic depth of the study.

Several limitations should be acknowledged. Given that this study examined a single AI implementation at a single site within one health care system, the transferability of our findings may be limited in settings with different organizational structures, radiology workflows, or AI technologies. The study was also conducted during early implementation and therefore did not capture how clinicians’ perceptions and determinants of adoption may evolve with prolonged use. Participants may also have moderated their responses because interviews were conducted face-to-face by a physician in their workplace, and one investigator was involved in the program’s implementation. As participant allocation to IDIs and FGDs was nonrandom, the data collection method may have influenced how participants expressed their views. However, the subthemes were represented across both IDIs and FGDs, including the socially mediated themes of peer influence and institutional trust. Finally, as a qualitative study, it did not assess objective clinical or safety outcomes of the program.

### Implications for Practice and Future Research

Our findings underscore the importance of a context-sensitive approach to AI implementation in primary care. Before rollout, implementation leaders could clearly communicate the clinical and operational problem that the AI intervention is intended to address, how it integrates into existing workflows, and its locally validated performance and limitations. This may facilitate a clearer understanding of the rationale for change among end users [25,38]. Beyond this, implementation strategies could consider how local social and organizational dynamics may influence adoption. Where appropriate, approaches such as peer champions could be used to model use, shape norms, and build confidence [26], alongside feedback loops to refine workflows and sustain engagement through a stepwise approach. Finally, the diversity of clinicians’ views in this study reflects heterogeneous barriers, suggesting that one-size-fits-all implementation strategies may not be effective and that a more personalized approach may be needed [27,39].

While these overarching implementation principles may be transferable across settings, the specific barriers encountered may differ according to the AI intervention, its integration into clinical workflows, and the organizational, cultural, and medicolegal context. Implementation strategies should therefore be adapted accordingly. In our setting, this could include accounting for differences in clinicians’ confidence in CXR interpretation and the influence of peer and institutional factors. Specific concerns about AI use could be addressed through targeted strategies such as training on the tool’s intended role and limitations, ongoing performance auditing and monitoring, and clear guidance on clinical accountability and human oversight.

A comprehensive evaluation of this AI intervention could integrate qualitative findings with objective clinical and operational outcomes, including diagnostic accuracy, radiologist reporting turnaround time, clinical decision-making time, workflow efficiency, and safety metrics. Patient-reported experience measures could also explore the intervention’s impact on patients’ experience of waiting and care delivery. Later-stage implementation outcomes, such as penetration, sustainability, and cost-effectiveness, could also be examined [13]. Longitudinal qualitative research could examine how clinicians’ perceptions of AI and its integration into clinical workflows evolve with prolonged routine use. Studies across other health care settings and workflow designs would also help determine whether similar patterns of adoption emerge elsewhere.

### Conclusion

This qualitative study suggests that adoption of AI-supported CXR triage in primary care is shaped not only by the technology itself, but also by how it is introduced, understood, and experienced in practice. Clinicians described greater willingness to use the program when they understood its purpose, found it useful within existing workflows, and trusted the organizational safeguards surrounding its use. These findings support the need for implementation strategies that are responsive to end user perspectives, local workflows, and the realities of primary care practice when integrating AI into routine care.

## Supplementary material

10.2196/103006 Multimedia Appendix 1 Semistructured interview guide.

10.2196/103006 Checklist 1 COREQ checklist.

## Abbreviations

COREQ: Consolidated Criteria for Reporting Qualitative Research
CXR: chest X-ray
FGD: focus group discussion
IDI: in-depth interview
NHG: National Healthcare Group
TAM3: Technology Acceptance Model 3
TDF: Theoretical Domains Framework

## References

[1] Hosny A, Parmar C, Quackenbush J, Schwartz LH, Aerts H (2018). Artificial intelligence in radiology. Nat Rev Cancer.

[2] National Healthcare Group Diagnostics.

[3] Goh CXY, Ho FCH (2023). The growing problem of radiologist shortages: perspectives from Singapore. Korean J Radiol.

[4] Ang A (2024). National Healthcare Group adopts AI for lung, heart disease screening. Healthcare IT News.

[5] Hwang EJ, Park S, Jin KN (2019). Development and validation of a deep learning-based automated detection algorithm for major thoracic diseases on chest radiographs. JAMA Netw Open.

[6] van Beek EJR, Ahn JS, Kim MJ, Murchison JT (2023). Validation study of machine-learning chest radiograph software in primary and emergency medicine. Clin Radiol.

[7] Miró Catalina Q, Vidal-Alaball J, Fuster-Casanovas A, Escalé-Besa A, Ruiz Comellas A, Solé-Casals J (2024). Real-world testing of an artificial intelligence algorithm for the analysis of chest X-rays in primary care settings. Sci Rep.

[8] Schalekamp S, van Leeuwen K, Calli E (2024). Performance of AI to exclude normal chest radiographs to reduce radiologists’ workload. Eur Radiol.

[9] Speets AM, van der Graaf Y, Hoes AW (2006). Chest radiography in general practice: indications, diagnostic yield and consequences for patient management. Br J Gen Pract.

[10] Shin HJ, Han K, Ryu L, Kim EK (2023). The impact of artificial intelligence on the reading times of radiologists for chest radiographs. NPJ Digit Med.

[11] Shin HJ, Lee S, Kim S, Son NH, Kim EK (2023). Hospital-wide survey of clinical experience with artificial intelligence applied to daily chest radiographs. PLoS One.

[12] Sim JZT, Lin J, Fong QW (2026). How threshold customisation affects the performance of a multiclass X-ray AI model for primary care triage: a retrospective study. BMJ Open.

[13] Proctor E, Silmere H, Raghavan R (2011). Outcomes for implementation research: conceptual distinctions, measurement challenges, and research agenda. Adm Policy Ment Health.

[14] Greenhalgh T, Robert G, Macfarlane F, Bate P, Kyriakidou O (2004). Diffusion of innovations in service organizations: systematic review and recommendations. Milbank Q.

[15] Atkins L, Francis J, Islam R (2017). A guide to using the Theoretical Domains Framework of behaviour change to investigate implementation problems. Implement Sci.

[16] Allen MR, Webb S, Mandvi A, Frieden M, Tai-Seale M, Kallenberg G (2024). Navigating the doctor-patient-AI relationship - a mixed-methods study of physician attitudes toward artificial intelligence in primary care. BMC Prim Care.

[17] Buck C, Doctor E, Hennrich J, Jöhnk J, Eymann T (2022). General practitioners’ attitudes toward artificial intelligence-enabled systems: interview study. J Med Internet Res.

[18] Saw SN, Ng KH (2022). Current challenges of implementing artificial intelligence in medical imaging. Phys Med.

[19] Scipion CEA, Manchester MA, Federman A, Wang Y, Arias JJ (2025). Barriers to and facilitators of clinician acceptance and use of artificial intelligence in healthcare settings: a scoping review. BMJ Open.

[20] Thanthrige A, Lu B, Sako Z, Wickramasinghe N (2025). Determinants of health care technology adoption using an integrated unified theory of acceptance and use of technology and task technology fit model: systematic review and meta-analysis. J Med Internet Res.

[21] Sandelowski M (2000). Whatever happened to qualitative description?. Res Nurs Health.

[22] Venkatesh V, Bala H (2008). Technology acceptance model 3 and a research agenda on interventions. Decision Sciences.

[23] Braun V, Clarke V (2006). Using thematic analysis in psychology. Qual Res Psychol.

[24] Lincoln YS, Guba EG (1985). Naturalistic Inquiry.

[25] Murray E, Treweek S, Pope C (2010). Normalisation process theory: a framework for developing, evaluating and implementing complex interventions. BMC Med.

[26] Dedehayir O, Ortt RJ, Riverola C, Miralles F (2017). Innovators and early adopters in the diffusion of innovations: a literature review. Int J Innov Mgt.

[27] Grayson ML, Macesic N, Huang GK (2015). Use of an innovative personality-mindset profiling tool to guide culture-change strategies among different healthcare worker groups. PLoS One.

[28] Vasilev Y, Vladzymyrskyy A, Omelyanskaya O, Blokhin I, Kirpichev Y, Arzamasov K (2023). AI-based CXR first reading: current limitations to ensure practical value. Diagnostics (Basel).

[29] Lind Plesner L, Müller FC, Brejnebøl MW (2023). Commercially available chest radiograph AI tools for detecting airspace disease, pneumothorax, and pleural effusion. Radiology.

[30] Masawi TJ, Miller E, Rees D, Thomas R (2025). Clinical perspectives on AI integration: assessing readiness and training needs among healthcare practitioners. Journal of Decision Systems.

[31] Laï MC, Brian M, Mamzer MF (2020). Perceptions of artificial intelligence in healthcare: findings from a qualitative survey study among actors in France. J Transl Med.

[32] Hofstede G, Hofstede GJ, Minkov M (2010). Cultures and Organizations: Software of the Mind: Intercultural Cooperation and Its Importance for Survival.

[33] Blease C, Kaptchuk TJ, Bernstein MH, Mandl KD, Halamka JD, DesRoches CM (2019). Artificial Intelligence and the future of primary care: exploratory qualitative study of UK general practitioners’ views. J Med Internet Res.

[34] Khullar D, Casalino LP, Qian Y, Lu Y, Chang E, Aneja S (2021). Public vs physician views of liability for artificial intelligence in health care. J Am Med Inform Assoc.

[35] Ministry of Health Singapore (2026). Healthcare Services Act. Healthcare services (clinical laboratory service and radiological service) regulations 2021. Singapore Statutes Online.

[36] Mehrotra P, Bosemani V, Cox J (2009). Do radiologists still need to report chest x rays?. Postgrad Med J.

[37] Fazakarley CA, Breen M, Leeson P, Thompson B, Williamson V (2023). Experiences of using artificial intelligence in healthcare: a qualitative study of UK clinician and key stakeholder perspectives. BMJ Open.

[38] Wong Q, Lacombe M, Keller R, Joyce T, OʼMalley K (2019). Leading change with ADKAR. Nurs Manage.

[39] Cabana MD, Rand CS, Powe NR (1999). Why don’t physicians follow clinical practice guidelines? A framework for improvement. JAMA.

