# Supplementary material for: Primary Care Doctors’ Perspectives and Experiences With a Chest X-Ray AI Triage Program: Qualitative Study
Source: J Med Internet Res. 2026 Sep 17;28:e103006. doi: 10.2196/103006 (PMC13585305; doi:10.2196/103006)
Supplement: Supplementary file 1 [file jmir-v28-e103006-s002.docx]

**Multimedia Appendix 2: COREQ Checklist**
