# Supplementary material for: Primary Care Doctors’ Perspectives and Experiences With a Chest X-Ray AI Triage Program: Qualitative Study
Source: J Med Internet Res. 2026 Sep 17;28:e103006. doi: 10.2196/103006 (PMC13585305; doi:10.2196/103006)
Supplement: Supplementary file 2 [file jmir-v28-e103006-s001.doc]

**Multimedia Appendix 1: Semi-Structured Interview Guide**

| **Interview guide** | | |
| --- | --- | --- |
| **Main questions** | | **Prompts** |
| **Opening questions** | | |
| 1 | Tell me about your experiences with interpretation of CXR in your practice. | - How frequently do you interpret CXR? - How do you usually interpret the CXR? Do you look at the CXR images and radiologist report or radiologist report only? - What are some challenges that you encountered? |
| **Section 1: Acceptability** | | |
| 2 | What do you think of the AI report? | - What was your initial reaction to seeing the report?   (Content)   - What are your thoughts about the content of the AI report? What else would you like to see in the AI report?   (Delivery)   - How easy was it to understand the report? How about the heat map? Could you explain why you thought so. - How easy was it to access the report? - How intuitive was the user interface? |
| 3 | What do you think of the AI interpretation of the CXRs? | (Credibility)   - How credible do you think the report is? - Do you trust the AI interpretation? - How would you approach situations where your interpretation of the CXR differs from the AI's? |
| 4 | How comfortable are you with using the report? | (Comfort)   - How much effort does it take to use it? |
| **Section 2: Adoption** | | |
| 5 | Would you adopt this AI program into your daily practice? |  |
| 6 | What factors would affect your decision? | Intrinsic factors   - Perceived competence at interpreting CXR - Self-efficacy and desire to be empowered to read CXR - Computer playfulness and affinity with technology, computer anxiety, perceived enjoyment - Beliefs about consequences such as impact on workload, patient outcomes, medicolegal implications, physician well-being (stress, burn-out) - Perceived alignment with professional role and expectations   Extrinsic factors   - Subjective norm/ social influences, organizational norms, commitment to organization - Perceptions of external control – the extent doctors believe that there are resources to support the use of the AI program, including manpower resources, training, technical support   AI program   - Experience - Voluntariness (How do you think your attitude towards the AI program would change if its use was mandatory?) |
| **Section 3: Appropriateness** | | |
| 7 | How have you used the AI program so far? | - How can the AI program be best used? - What are the scenarios in which the AI program is the most, or least, useful? - Are there situations where you might not use it? - How can the AI program be of benefit to staff, patients, and organization? |
| 8 | How can the AI program fit into your current practice? | - Did you need to make adjustments to accommodate the AI tool in your practice? If so, how? (E.g., referral practices, timeliness of care) - How has the AI program aligned with your professional identity and boundaries as a primary care doctor? |
| **Section 4: Feasibility** | | |
| 9 | How often do you use the AI program in daily practice? |  |
| 10 | What practical issues have you encountered when you use the AI program? | Examples   - Technical challenges - Workload pressures - Manpower limitations - Workflow disruptions - Training inadequacy |
| 11 | What resources would be helpful to you? | - How has the support been thus far? - How do you feel about the technical support? - What are your thoughts about the training? - What other skills might you need to develop? |
| 12 | How would you feel if you were to interpret the CXR on your own, with the help of the AI report? | - Would you be comfortable without a radiologist’s report? In what situations would you be comfortable?   How would you feel if   - The AI tool is used as triage such that CXRs labelled as “normal” by AI are not reported by radiologists on the same day - The AI tool is used as triage such that all CXRs are not reported by radiologists on the same day except those labelled “critically abnormal” by AI - No CXRs are reported by radiologists   If any of the above workflows are feasible to you, how can they be implemented to minimise the practical issues mentioned? |
| **Section 5: Fidelity** | | |
| 13 | What are your thoughts on using the AI program as intended? |  |
| **Closing questions** | | |
| 14 | Is there anything else you would like to add about the use of the AI program in primary care? |  |
